# Childhood Cancer and Traffic-Related Air Pollution Exposure in Pregnancy and Early Life

**DOI:** 10.1289/ehp.1306761

**Published:** 2013-09-10

**Authors:** Julia E. Heck, Jun Wu, Christina Lombardi, Jiaheng Qiu, Travis J. Meyers, Michelle Wilhelm, Myles Cockburn, Beate Ritz

**Affiliations:** 1 Department of Epidemiology, School of Public Health, University of California, Los Angeles, Los Angeles, California, USA; 2 Program in Public Health and Department of Epidemiology, University of California, Irvine, Irvine, California, USA; 3 Department of Biostatistics, School of Public Health, University of California, Los Angeles, Los Angeles, California, USA; 4 Department of Preventive Medicine, Keck School of Medicine, University of Southern California, Los Angeles, California, USA

## Abstract

Background: The literature on traffic-related air pollution and childhood cancers is inconclusive, and little is known on rarer cancer types.

Objectives: We sought to examine associations between childhood cancers and traffic-related pollution exposure.

Methods: The present study included children < 6 years of age identified in the California Cancer Registry (born 1998–2007) who could be linked to a California birth certificate (*n* = 3,590). Controls were selected at random from California birthrolls (*n* = 80,224). CAlifornia LINE Source Dispersion Modeling, version 4 (CALINE4) was used to generate estimates of local traffic exposures for each trimester of pregnancy and in the first year of life at the address indicated on the birth certificate. We checked our findings by additionally examining associations with particulate matter (≤ 2.5 μm in aerodynamic diameter; PM_2.5_) pollution measured by community-based air pollution monitors, and with a simple measure of traffic density.

Results: With unconditional logistic regression, a per interquartile range increase in exposure to traffic-related pollution during the first trimester (0.0538 ppm carbon monoxide, estimated using CALINE4) was associated with acute lymphoblastic leukemia [ALL; first trimester odds ratio (OR) = 1.05; 95% CI: 1.01, 1.10]; germ cell tumors (OR = 1.16; 95% CI: 1.04, 1.29), particularly teratomas (OR = 1.26; 95% CI: 1.12, 1.41); and retinoblastoma (OR = 1.11; 95% CI: 1.01, 1.21), particularly bilateral retinoblastoma (OR = 1.16; 95% CI: 1.02, 1.33). Retinoblastoma was also associated with average PM_2.5_ concentrations during pregnancy, and ALL and teratomas were associated with traffic density near the child’s residence at birth.

Conclusions: We estimated weak associations between early exposure to traffic pollution and several childhood cancers. Because this is the first study to report on traffic pollution in relation to retinoblastoma or germ cell tumors, and both cancers are rare, these findings require replication in other studies.

Citation: Heck JE, Wu J, Lombardi C, Qiu J, Meyers TJ, Wilhelm M, Cockburn M, Ritz B. 2013. Childhood cancer and traffic-related air pollution exposure in pregnancy and early life. Environ Health Perspect 121:1385–1391; http://dx.doi.org/10.1289/ehp.1306761

## Introduction

Motor vehicle emissions are a major source of ambient air pollution in the United States and elsewhere. In a recent meeting, the International Agency for Research on Cancer (IARC) classified diesel exhaust as carcinogenic and gasoline exhaust as possibly carcinogenic to humans (Benbrahim-Tallaa et al. 2012). Traffic exhaust contains carbon monoxide, nitrogen oxides, and toxic air contaminants such as benzene, formaldehyde, 1,3-butadiene, and nitroarenes. Particulate components of traffic exhaust include metals, elemental carbon, organic carbon, and sulphate. A number of these components have been classified as established or suspected carcinogens in occupational settings (IARC 2012).

The literature on traffic-related air pollution and childhood cancers has been equivocal, likely for several reasons, including variation in exposure assessment methods and time periods of exposure. In addition, because of small numbers of cases, disparate cancer types were grouped as a single outcome. Many studies have used simple proxy measures of exposure such as rates of neighborhood car ownership, gas station density, or residential proximity to roads, gas stations, or car repair shops (Abdul Rahman et al. 2008; Alexander et al. 1996; Brosselin et al. 2009; Harrison et al. 1999; Nordlinder and Jarvholm 1997; Reynolds et al. 2002; Steffen et al. 2004; Weng et al. 2009). Other studies have classified exposure based on traffic density (Harrison et al. 1999; Langholz et al. 2002; Pearson et al. 2000; Reynolds et al. 2001, 2002, 2004; Savitz and Feingold 1989; Visser et al. 2004; Von Behren et al. 2008). Only a few studies have classified exposure based on measurements of air pollutants from air monitors (Amigou et al. 2011; Weng et al. 2008) or sophisticated air pollution modeling strategies that consider more factors that influence exhaust levels such as chemical reactions of pollutants, background pollution levels, land use, or weather (Crosignani et al. 2004; Feychting et al. 1998; Raaschou-Nielsen et al. 2001; Vinceti et al. 2012). In a previous study that compared different ways of measuring traffic-related air pollution exposures in relation to birth outcomes, Wu et al. (2011) showed that traffic density yields lower effect estimates than those generated in more complex models.

The literature is also limited in scope because most studies have reported only on leukemias, central nervous system (CNS) tumors, or all childhood cancer types combined, and few have had sufficient sample sizes to stratify by cancer subtypes or estimate associations with rarer tumors. Further, most studies assessed exposure using the child’s address at the time of diagnosis, study entry, or death, and are therefore best interpreted as estimating associations with traffic exposure during childhood. Because the pathogenesis of at least some childhood cancers is likely to begin *in utero*, these studies may not capture an important exposure period for early childhood cancers (Greaves and Wiemels 2003; Lafiura et al. 2007).

We *a priori* hypothesized that because of the fetus’s greater vulnerability to environmental toxins, exposures during the pregnancy period would be most relevant for childhood cancer risk (Selevan et al. 2000). To our knowledge, six studies have examined associations between childhood cancers and exposures during pregnancy. Two reported that living near gas stations or auto repair garages was associated with acute lymphoblastic leukemia (ALL) and acute myeloid leukemia (AML) (Brosselin et al. 2009) or with all leukemias combined (Steffen et al. 2004). Of studies that evaluated traffic density near the child’s residence, two reported no association with leukemias (Reynolds et al. 2001, 2004), and a third reported no association with ALL specifically (Von Behren et al. 2008). However, traffic density was associated with CNS tumors in the Reynolds et al. (2004) study. One study that used Operational Street Pollution Modelling, which examined children who were diagnosed up through 15 years of age, found no associations between traffic pollution and leukemias, CNS tumors, or lymphomas, except that associations were observed between Hodgkin lymphoma and both benzene and modeled nitrogen dioxide (NO_2_) as a marker of traffic, modeled as continuous variables (Raaschou-Nielsen et al. 2001).

The purpose of the present study was to estimate associations between common childhood cancers and traffic-related air pollution exposure during pregnancy and early childhood (< 1 year of age).

## Methods

In the present investigation we identified incident cases of cancers from the California Cancer Registry diagnosed 1998–2007 (born 1998–2007) (Heck et al. 2013a). Because our main interest was exposures occurring in pregnancy and early life, we included only cases ≤ 5 years of age at the time of cancer diagnosis. We used a probabilistic linkage program (LinkPlus; CDC, Atlanta, GA) to match each registry case with his or her birth certificate by first and last names, dates of birth, and (when available) social security number; we were able to match 89% of cases to a California birth certificate. Controls, frequency-matched by year of birth (20:1 matching rate), were randomly selected from California birth rolls. Controls had no record of a cancer diagnosis in California before 6 years of age. Because this was a noncontact study, it was deemed exempt from informed consent requirements. All information was taken from Cancer Registry records, birth certificates, or year 2000 Census data. Strict provisions were made to maintain the confidentiality of participants. The study received approvals from the human subjects protection boards of the University of California, Los Angeles; the University of California, Irvine; and the California Health and Human Services Agency.

At all time points, air pollution exposure was estimated based upon each child’s address as listed on the birth certificate. Addresses were automatically geocoded using our open-source geocoder with a manual resolution process for unmatched addresses (Goldberg et al. 2008). Resulting locations were recorded along with the relevant date of birth and gestational age information, as taken from the birth certificate. When gestational ages were improbably long (> 45 weeks), they were classified as missing. We defined the first trimester as weeks 1–13 of pregnancy, the second trimester as weeks 14–26 of pregnancy, and the third trimester as week 27 to birth.

The present study included only children for whom there was parcel-level or street-level geocoding and who listed a home address within California (*n* = 84,268). Of these, four children were excluded because their addresses were located on the islands off the Los Angeles coast with no roadway data available. We report on cancer subtypes for which there were at least 65 cases, as classified by SEER (Surveillance, Epidemiology, and End Results) recode of *International Classification of Childhood Cancer, 3rd Edition* (ICCC-3; Steliarova-Foucher et al. 2005). The final analysis included 3,590 cases [1,280 cases of ALL (SEER code 011), 229 AML (code 012), 78 non-Hodgkin lymphomas (NHL; code 022–023), 125 ependymomas (code 031), 282 astrocytomas (code 032), 205 intracranial and intraspinal embryonal tumors (code 033), 99 medulloblastomas [*International Classification of Diseases for Oncology, 3rd Edition* (ICD-O) (Fritz 2000) codes 9470–9472, 9474, 9480], 65 primitive neuroectodermal tumors (PNET; ICD-O-3 code 9473), 417 neuroblastomas (code 041), 254 retinoblastomas (code 050; 165 unilateral, 87 bilateral, and 2 with laterality unknown), 298 Wilms tumors (code 061), 127 hepatoblastomas (code 071), 144 rhabdomyosarcomas (code 091), 140 germ cell tumors [codes 101-105; including 72 teratomas (ICD-O-3 code 9080), 49 yolk sac tumors (ICD-O-3 code 9071), and 19 of other histologic types], and 80,224 controls.

CAlifornia LINE Source Dispersion Model, version 4 (CALINE4) is a Gaussian dispersion model used to quantify exposure to traffic pollutants at a given address. Previous studies have found moderate to high correlations (*r* = 0.55–0.95) of CALINE4-modeled estimates with measured variability of traffic-related air pollutants (Benson 1989, 1992; Broderick et al. 2005; Gauderman et al. 2005; Levitin et al. 2005). Model inputs were based on local traffic emissions of both gasoline vehicles and diesel trucks within a 1,500-m buffer around an address, and included traffic volumes, roadway geometry, vehicle emission rates, and meteorology. We selected the 1,500-m buffer because previous studies showed narrow impact zones of primary traffic emissions, ranging from 300 m (Zhu et al. 2002) to up to 2,600 m (Hu et al. 2009). The California Department of Transportation (Caltrans) provided information on annual average daily traffic counts on freeways, highways, and “major surface streets,” as defined by Caltrans. Annual average daily truck traffic counts on freeways and highways were also obtained from Caltrans. Emission factors for carbon monoxide (CO), nitrogen oxides (NO_x_), and particulate matter with aerodynamic diameter ≤ 2.5 μm (PM_2.5_)were obtained by county, year, and season (winter and summer) from the California Air Resources Board (CARB)’s emission factors (EMFAC2007) vehicle emissions model (CARB 2007). Hourly wind speed, direction, and temperature data were obtained from the CARB Air Quality and Meteorological Information System (CARB 2010). Our validation of the CALINE4 model showed a high correlation (*r* = 0.87) of CALINE4-modeled monthly NO_x_ concentrations with measurements at nine monitoring sites in the Long Beach study area in 2007 and 2008 (unpublished data).

CALINE4 modeling produces average estimates of local long-term exposure to CO, NO_x_, and PM_2.5_. These pollutants should be considered as markers for the traffic-related pollution mix rather than as carcinogens in and of themselves. Values were computed for each of the four time periods of interest (first through third trimesters and first year of life). Because correlations between modeled CO and NO_x_ (*r* ~ 0.98–0.99) and between PM_2.5_ and both CO and NO_x_ (*r* ~ 0.90–0.93) were very high, we report results only for modeled CO.

Numbers of children included in analyses varied according to the time period examined. Children born before the third trimester were excluded from analyses of exposures during the third trimester, along with 4,961 children (217 cases, 4,744 controls) with missing information on gestational age. Children who died during their first year of life (485 cases, 467 controls) were excluded from analyses of exposures during the first year. In addition, if meteorological values were missing for more than 25% of days during any time period for a given child, the child was excluded from the analysis of that time period (1.7–3.6% of participants across the four time periods).

Associations between air pollution and childhood cancers were estimated using separate unconditional logistic regression models (SAS Institute Inc., Cary, NC) for each outcome, using the same group of 80,224 controls in each regression. We estimated increases in odds of each cancer per interquartile range (IQR) increase in CO. We adjusted for potential confounders using data acquired from birth certificates and year 2000 census information. Confounding variables included in multivariate models were the child’s year of birth, mother’s and father’s race/ethnicity [non-Hispanic white, non-Hispanic black, Hispanic (any race), or other/not specified], mother’s education (≤ 8, 9–< 12, 12, 13–15, or ≥ 16 years), mother’s country of birth (United States, Mexico, or other), parity (0, 1, ≥ 2), method of payment for prenatal care (private insurance vs. other payment methods, including self-pay and government programs), and neighborhood socioeconomic index. Both race/ethnicity and socioeconomic status are related to traffic pollution exposure in California (Houston et al. 2004). Neighborhood socioeconomic index was derived using principal components analysis to create a single, five-level socioeconomic status measure from seven census tract–level indicators of socioeconomic status (education, median household income, percent living 200% below poverty, percent blue-collar workers, percent older than 16 years without employment, median rent, and median house value) (Yost et al. 2001). We have previously shown that method of payment for prenatal care is a reasonable proxy for family income (Ritz et al. 2007). Parity (Dockerty et al. 2001; Schuz et al. 1999; Shu et al. 1995) and mother’s country of birth (Heck et al. 2012; Johnson et al. 2008; Von Behren and Reynolds 2003) have previously been reported to be associated with childhood cancers. We considered adjustment for maternal age and paternal years of education, but because their inclusion in models did not change effect estimates by 10%, they were left out of the final models. Father’s country of birth is not collected on California birth certificates.

We additionally examined associations between cancers and average PM_2.5_ concentrations measured at stations during the entire pregnancy. For these analyses, we restricted our study population to children with addresses at birth that were within 5 miles (^~^_−⊇_8 km) of a CARB air monitoring station. The distance from each participant’s home to each air-monitoring station was assessed using ArcView GIS software (version 3.3; ESRI, Redlands, CA). Average concentrations during pregnancy were based on hourly and 24-hr average measurements of PM_2.5_, with most stations recording measurements every 3 days.

We also estimated associations with traffic density, based on 2002 average annual daily traffic data. Traffic densities were calculated on a 20 × 20 m grid using the kernel density plotting feature of Spatial Analyst in ArcInfo GIS 9.1 (ESRI), which effectively caused the densities to decrease from volume-dependent values at roadway edges to zero at 500-m perpendicular distance from roadways. The 500-m distance was chosen for consistency with previous studies (Reynolds et al. 2001, 2004), but we also estimated associations with traffic density using 300-m and 1,000-m buffers.

For ALL cases, we conducted stratified analyses to determine whether associations differed by the age at diagnosis (< 1 year or ≥ 1 year). Because one-third of California births occur in Los Angeles County, we also conducted sensitivity analyses to determine whether associations with ALL differed between children born in Los Angeles County versus the rest of the state. We also estimated associations separately for the two most common histologic subtypes of germ cell tumors (yolk sac tumors and teratomas). Finally, we estimated associations with quartiles of CO exposure during each pregnancy period, which were defined based on the exposure distribution among controls. All regression analyses included the covariates listed above.

## Results

We report sociodemographic characteristics of cases and controls in Table 1. There were differences from controls for each covariate in relation to at least one of the cancer outcomes assessed. Maternal years of education was missing for 2% of the sample, with no differences in the amount of missing by case status; all other covariates had < 1% missing data.

**Table 1:** Demographic and socioeconomic characteristics of cases and controls.

Characteristic	Controls	ALL	AML	NHL	Epen-dymoma	Astro-cytomas	Intracranial and intraspinal embryonal tumors	Medullo-blastoma	PNET	Neuro-blastoma	Retino-blastoma	Wilms tumor	Hepato-blastoma	Rhabdomyo-sarcoma	Germ cell tumors
*n*	80,224	1,280	229	78	125	282	205	99	65	417	254	298	127	144	140
Mother’s race/ethnicity (%)
Non-Hispanic white	30.6	30.7*	30.1	42.3	34.4	42.2*	36.6	35.4	32.3	42.2*	29.1	37.2*	34.6	29.9	25.7*
Hispanic	50.0	54.5	53.3	46.2	52.0	42.9	45.4	47.5	49.2	39.8	50.4	45.6	50.4	46.5	47.9
Non-Hispanic black	6.1	2.9	4.8	2.6	5.6	5.0	5.9	6.1	7.7	5.5	5.5	9.1	3.9	8.3	5.0
Other/not specified	13.3	11.9	11.8	9.0	8.0	9.9	12.2	11.1	10.8	12.5	15.0	8.1	11.0	15.3	21.4
Mother’s years of education (%)
≤ 8	11.4	11.7	16.5	4.0	8.2	8.6	6.9	4.1	14.1	8.4*	5.3*	9.5*	17.5	9.9	15.4
9–< 12	18.4	17.1	15.2	28.0	18.0	14.4	20.2	24.5	18.8	14.0	21.5	13.3	15.9	16.2	16.9
12	28.3	30.4	29.9	29.3	28.7	28.1	26.1	29.6	23.4	29.7	30.1	34.7	26.2	27.5	30.1
13–15	19.6	18.3	17.0	14.7	23.0	22.3	21.7	20.4	17.2	19.9	22.8	18.7	19.0	19.7	19.1
≥ 16	22.2	22.6	21.4	24.0	22.1	26.6	25.1	21.4	26.6	28.0	20.3	23.8	21.4	26.8	18.4
Mother’s birthplace (%)
Mexico	27.6	28.2	30.1	20.5	22.6	21.7*	20.5	20.2	23.1	20.1*	22.0	22.1*	29.9	27.1	29.3
Other foreign	18.9	16.6	20.1	15.4	14.5	15.7	22.9	22.2	26.2	18.0	19.3	14.4	16.5	21.5	24.3
USA	53.5	55.2	49.8	64.1	62.9	62.6	56.6	57.6	50.8	61.9	58.7	63.4	53.5	51.4	46.4
Parity (%)
0	39.0	36.0	40.6*	43.6	36.0	45.4*	45.4	51.5*	36.9	41.0	40.6*	40.3*	38.6	37.5	32.9
1	32.0	32.7	24.9	30.8	31.2	31.6	31.2	25.3	43.1	34.5	25.2	26.5	32.3	34.7	40.0
≥ 2	28.9	31.3	34.5	25.6	32.8	23.0	23.4	23.2	20.0	24.5	34.3	33.2	29.1	27.8	27.1
Father’s race/ethnicity (%)
Non-Hispanic white	29.2	31.8*	25.8	34.6	30.4	40.1*	36.6	30.3	35.4	38.1*	28.3	37.9*	33.9	31.3	25.0
Hispanic of any race	46.6	49.8	52.4	48.7	49.6	40.4	40.5	43.4	43.1	38.8	44.9	43.6	49.6	44.4	46.4
Non-Hispanic black	6.6	3.4	6.6	2.6	7.2	6.7	6.8	7.1	7.7	6.5	5.9	8.1	4.7	6.9	4.3
Other/not specified	17.5	15.0	15.3	14.1	12.8	12.8	16.1	19.2	13.8	16.5	20.9	10.4	11.8	17.4	24.3
Source of payment for prenatal care (%)
Private insurance, HMO	52.6	57.2*	51.5	60.3	63.6*	64.5*	57.8	53.5	51.6	58.6*	55.7	59.7*	50.4	59.9	52.9
Medi-Cal/other govt/self-pay	47.4	42.8	48.5	39.7	36.4	35.5	42.2	46.5	48.4	41.4	44.3	40.3	49.6	40.1	47.1
Neighborhood socioeconomic index (%)
1 (low)	27.9	27.6	28.8*	28.2	21.6	24.5	23.4	24.2	27.7	23.7	30.7	24.5	28.3	29.2	19.3
2	23.7	23.0	27.9	28.2	29.6	23.0	28.3	34.3	20.0	24.5	22.0	24.5	16.5	19.4	29.3
3	19.3	20.2	15.7	11.5	21.6	18.4	18.0	14.1	23.1	20.9	20.1	20.1	17.3	22.9	22.9
4	15.4	15.2	14.4	21.8	12.0	17.7	16.6	13.1	20.0	15.8	13.8	17.4	17.3	11.8	15.0
5 (high)	13.7	13.9	12.7	10.3	15.2	16.3	13.7	14.1	9.2	15.1	13.4	13.4	20.5	16.7	13.6
govt, government. *p < 0.05 from chi-square testing.															

Across the time periods, modeled CO values were highly correlated (Table 2). Correlations were weaker between CALINE4 CO measures and both traffic density and PM_2.5_. We observed elevated odds for specific childhood cancers and traffic-related air pollutants and specific childhood cancers across gestation and in the first year of life; because of strong correlations across time periods, we do not present second- and and third-trimester results (Table 3). IQR increases of modeled CO were positively associated with ALL, retinoblastoma (overall and bilateral only), and germ cell tumors; increases in PNET, ependymomas, and NHL were also observed. Associations between CO and germ cell tumors appeared to be driven by associations with teratomas. We also estimated a negative association between AML and an IQR increase in CO during the first trimester.

**Table 2:** Descriptive statistics of air pollution measures and Pearson correlation coefficients across time periods.

Measure	n	Mean ± SD	IQR	CALINE4 modeled CO				PM_2.5_	Traffic density		
				1st trimester	2nd trimester	3rd trimester	Child’s 1st year	Average in pregnancy	Within 300 m	Within 500 m	Within 1,000 m
CALINE4-modeled CO (ppm)
1st trimester	82,411	0.0564 ± 0.0653	0.0538	1.00
2nd trimester	82,308	0.0555 ± 0.0644	0.0530	0.97	1.00
3rd trimester	77,167	0.0551 ± 0.0647	0.0528	0.96	0.98	1.00
1st year average	81,003	0.0548 ± 0.0633	0.0523	0.97	0.97	0.98	1.00
PM_2.5_(μg/m^3^)	56,548	17.21 ± 5.09	8.1	0.22	0.22	0.21	0.22	1.00
Traffic density (vehicle no./day × m/m^2^)
Within 300 m	83,814	65.18 ± 110.08	73.17	0.64	0.64	0.64	0.66	0.07	1.00
Within 500 m	83,814	71.05 ± 97.68	67.75	0.74	0.75	0.75	0.76	0.09	0.90	1.00
Within 1,000 m	83,814	75.53 ± 79.43	68.84	0.81	0.81	0.81	0.82	0.13	0.66	0.86	1.00
PM_2.5_ was measured at the closest community air monitor located within 5 miles of the child’s residence. Traffic density was calculated using traffic count data at major roads near the residence.											

**Table 3:** ORs (95% CIs) for cancer risk for 1 IQR increase in CO, using CALINE4 air pollution modeling.^*a*^

Outcome	*n* cases	Average traffic exposure in the 1st trimester of pregnancy		Average traffic exposure in the child’s first year	
		Model 1^*b*^	Model 2^*c*^	Model 1^*b*^	Model 2^*c*^
ALL	1,280	1.03 (0.99, 1.08)	1.05 (1.01, 1.10)	1.04 (0.99, 1.08)	1.05 (1.01, 1.10)
AML	229	0.87 (0.75, 1.00)	0.85 (0.73, 0.98)	0.94 (0.82, 1.08)	0.91 (0.79, 1.05)
NHL	78	1.07 (0.92, 1.25)	1.10 (0.95, 1.28)	1.06 (0.89, 1.25)	1.08 (0.92, 1.28)
Ependymoma	125	1.04 (0.90, 1.19)	1.08 (0.94, 1.24)	1.07 (0.94, 1.23)	1.11 (0.97, 1.27)
Astrocytoma	282	0.89 (0.80, 1.01)	0.94 (0.84, 1.06)	0.89 (0.79, 1.01)	0.95 (0.84, 1.06)
Intracranial and intraspinal embryonal tumors	205	0.99 (0.88, 1.11)	0.99 (0.87, 1.12)	1.01 (0.89, 1.14)	1.01 (0.89, 1.15)
Primitive neuroectodermal tumor	65	1.08 (0.91, 1.28)	1.04 (0.86, 1.25)	1.10 (0.93, 1.31)	1.07 (0.89, 1.29)
Medulloblastoma	99	0.94 (0.78, 1.12)	0.93 (0.77, 1.13)	0.95 (0.79, 1.14)	0.95 (0.79, 1.15)
Neuroblastoma	417	0.95 (0.87, 1.04)	0.98 (0.89, 1.07)	0.97 (0.89, 1.06)	1.00 (0.91, 1.09)
Retinoblastoma	254	1.10 (1.01, 1.20)	1.11 (1.01, 1.21)	1.11 (1.02, 1.22)	1.12 (1.02, 1.23)
Unilateral	165	1.07 (0.95, 1.20)	1.08 (0.96, 1.22)	1.07 (0.95, 1.21)	1.08 (0.95, 1.22)
Bilateral	87	1.15 (1.01, 1.31)	1.16 (1.02, 1.33)	1.18 (1.04, 1.34)	1.19 (1.05, 1.36)
Wilms tumor	298	0.88 (0.78, 0.99)	0.91 (0.81, 1.03)	0.88 (0.78, 0.99)	0.91 (0.81, 1.03)
Hepatoblastoma	127	0.94 (0.80, 1.12)	0.95 (0.80, 1.13)	0.98 (0.83, 1.15)	0.98 (0.82, 1.16)
Rhabdomyosarcoma	144	0.95 (0.82, 1.11)	0.91 (0.77, 1.08)	0.95 (0.81, 1.11)	0.90 (0.75, 1.07)
Germ cell tumors	140	1.14 (1.02, 1.27)	1.16 (1.04, 1.29)	1.15 (1.03, 1.28)	1.17 (1.05, 1.31)
Yolk sac tumors	49	0.95 (0.72, 1.24)	0.92 (0.68, 1.24)	0.92 (0.68, 1.23)	0.89 (0.64, 1.23)
Teratomas	72	1.24 (1.11, 1.39)	1.26 (1.12, 1.41)	1.25 (1.11, 1.41)	1.27 (1.13, 1.43)
^***a***^*n* controls = 80,224. ^***b***^ORs by unconditional logistic regression, adjusted only for birth year (matching variable). ^***c***^Additional adjustment for mother’s and father’s race/ethnicity, mother’s years of education, mother’s country of birth, parity, method of payment for prenatal care, and neighborhood socioeconomic index.					

In comparing results across California, the association between ALL and CO exposure during the child’s first year was similar for children residing in LA County [odds ratio (OR) = 1.06; 95% CI: 1.00, 1.12] and in the rest of the state (OR = 1.08; 95% CI: 0.99, 1.17). When ALL cases were stratified by age at diagnosis, we estimated stronger associations with cases diagnosed during the first year of life (OR = 1.14; 95% CI: 0.99, 1.31 based on 81 cases) than cases diagnosed at 1–5 years of age (OR = 1.04; 95% CI: 1.00, 1.09 based on 1,149 cases).

Associations with IQR increases in average PM_2.5_ exposures during pregnancy (7.84 μg/m^3^) and with an IQR increase in traffic density within 500 m of the residence at birth (68 vehicle number per day × meter/meter^2^) are shown in Table 4. Although sample sizes were smaller, the patterns observed in CALINE4 modeled CO were generally similar to associations with IQR increases in PM_2.5_, except that no association was observed between PM_2.5_ and germ cell tumors. Associations with traffic density tended to be closer to the null than those generated using CALINE4, regardless of the buffer distance used (results for 300 m and 1,000 m not shown).

**Table 4:** ORs (95% CIs) for childhood cancer from one IQR increase in traffic pollution exposure, measuring traffic-related air pollution exposure using PM_2.5_ measured from community air monitors, and traffic density.

Outcome	PM_2.5_ (*n* controls = 26,159)			Traffic density within 500 m of the residence (*n* controls = 77,892)		
	*n* cases	Model 1^*a*^	Model 2^*b*^	*n* cases	Model 1^*a*^	Model 2^*b*^
ALL	397	1.11 (0.94, 1.30)	1.10 (0.92, 1.30)	1,243	1.03 (0.99, 1.06)	1.03 (1.00, 1.07)
AML	82	0.93 (0.64, 1.35)	0.85 (0.57, 1.27)	224	0.89 (0.80, 1.00)	0.89 (0.79, 1.00)
NHL	28	0.75 (0.41, 1.38)	0.78 (0.41, 1.46)	75	1.05 (0.92, 1.21)	1.07 (0.93, 1.23)
Ependymoma	39	1.29 (0.72, 2.07)	1.26 (0.72, 2.21)	117	1.05 (0.94, 1.17)	1.07 (0.96, 1.20)
Astrocytoma	73	0.87 (0.60, 1.26)	0.90 (0.61, 1.34)	275	0.95 (0.87, 1.04)	0.98 (0.90, 1.08)
Intracranial and intraspinal embryonal tumors	68	1.06 (0.71, 1.57)	1.18 (0.78, 1.80)	202	1.00 (0.91, 1.10)	1.00 (0.91, 1.11)
Primitive neuroectodermal tumor	27	0.91 (0.49, 1.68)	1.05 (0.55, 2.00)	63	1.04 (0.90, 1.21)	1.03 (0.88, 1.21)
Medulloblastoma	29	1.61 (0.88, 2.95)	1.76 (0.91, 3.38)	98	0.93 (0.79, 1.09)	0.93 (0.78, 1.09)
Neuroblastoma	129	0.99 (0.74, 1.33)	1.04 (0.76, 1.41)	403	0.97 (0.90, 1.04)	0.99 (0.92, 1.07)
Retinoblastoma	87	1.57 (1.10, 2.23)	1.44 (0.99, 2.10)	245	1.05 (0.97, 1.13)	1.05 (0.97, 1.13)
Unilateral	65	1.60 (1.06, 2.41)	1.44 (0.93, 2.22)	162	1.01 (0.91, 1.12)	1.02 (0.92, 1.13)
Bilateral	22	1.48 (0.74, 2.95)	1.46 (0.70, 3.06)	81	1.10 (0.98, 1.23)	1.09 (0.97, 1.24)
Wilms tumor	100	1.06 (0.77, 1.47)	1.15 (0.82, 1.63)	294	0.97 (0.89, 1.05)	0.98 (0.90, 1.07)
Hepatoblastoma	42	0.79 (0.47, 1.33)	0.87 (0.49, 1.53)	126	0.86 (0.72, 1.01)	0.86 (0.73, 1.02)
Rhabdomyosarcoma	51	0.72 (0.45, 1.16)	0.80 (0.48, 1.33)	140	0.93 (0.81, 1.06)	0.90 (0.78, 1.04)
Germ cell tumors	41	0.87 (0.51, 1.47)	0.98 (0.56, 1.71)	136	1.10 (1.00, 1.20)	1.10 (1.00, 1.20)
Yolk sac tumors	12	0.91 (0.35, 2.40)	0.92 (0.34, 2.48)	46	0.98 (0.79, 1.20)	0.94 (0.75, 1.18)
Teratomas	23	0.73 (0.35, 1.49)	0.77 (0.36, 1.68)	71	1.17 (1.05, 1.30)	1.16 (1.04, 1.30)
^***a***^ORs by unconditional logistic regression, adjusted only for birth year (matching variable). ^***b***^Additional adjustment for mother’s and father’s race/ethnicity, mother’s years of education, mother’s country of birth, parity, method of payment for prenatal care, and neighborhood socioeconomic index.						

When CALINE4-based CO exposures across the time periods were categorized into quartiles, we observed similar patterns of findings to those seen when we conducted regression analyses based on the interquartile range (data not shown). For ALL, the point estimates were slightly increased across the quartiles relative to the lowest quartile (2nd quartile: OR = 1.07; 95% CI: 0.90, 1.27; 3rd quartile: OR = 1.14; 95% CI: 0.96, 1.35; highest quartile: OR = 1.12: 95% CI: 0.94, 1.34). For retinoblastoma, we estimated stronger ORs at the highest quartile (2nd quartile: OR = 1.10; 95% CI: 0.75, 1.62; 3rd quartile: OR = 1.12; 95% CI: 0.76, 1.66; highest quartile: OR = 1.50; 95% CI: 1.02, 2.19). Teratomas also exhibited higher ORs at the highest quartile (2nd quartile: OR = 1.27: 95% CI: 0.63, 2.56; 3rd quartile: OR = 0.90; 95% CI: 0.41, 1.98; highest quartile: OR = 2.10, 95% CI: 1.06, 4.16).

## Discussion

In this large population-based study, we observed positive associations of ALL, retinoblastoma, and teratomas with model-based estimates of traffic-related air pollution exposures during pregnancy and early childhood. Because of strong correlations of our modeled air pollutant exposure estimates across all three trimesters of pregnancy and the first year of life, we were not able to identify variation in associations according to time period. Leukemias have been studied at length in relation to traffic pollution, and our findings are consonant with what has been observed in most previous studies, which have showed increases in risk for leukemias with exposure both during pregnancy and in childhood (Amigou et al. 2011; Crosignani et al. 2004; Feychting et al. 1998; Vinceti et al. 2012; Weng et al. 2008). Although specific biological mechanisms underlying associations between air pollution and cancer are not known, experimental evidence indicates PM exposure instigates an immune response which increases TLR (toll-like receptor)(Shoenfelt et al. 2009) and RAGE (receptors for advanced glycation end-products) expression (Reynolds et al. 2011). These signaling pathways have been implicated in tumor growth and metastasis (Grimm et al. 2010; Sims et al. 2010).

Traffic-related air pollution exposure was positively associated with both unilateral and bilateral retinoblastoma, although associations with bilateral disease were stronger. Retinal abnormalities have been observed in neonates whose mothers smoked in pregnancy (Beratis et al. 2000), and CO, in higher doses, is known to cause retinal damage (Kelley and Sophocleus 1978; Resch et al. 2005). There have been few epidemiologic studies of retinoblastoma that examined environmental exposures. In one study, paternal occupational exposure to exhaust fumes was not related to increases in bilateral retinoblastoma, although the number of exposed cases was small (OR = 1.2; 95% CI: 0.6, 2.4) (MacCarthy et al. 2009).

Although teratomas were associated with traffic pollution when modeled using CALINE4 and traffic density, these associations were not replicated in our PM_2.5_-based analyses, suggesting that the association with teratomas might reflect an effect of agents in traffic exhaust that decay quickly over distances. Alternatively, this result may be due to noncausal mechanisms. Teratomas are believed to derive from primordial germ cells that arise in the yolk sac and migrate along the mesentery to the gonadal ridge during the 4th–5th week of development. Cells that incompletely differentiate are hypothesized to give rise to teratomas (Barksdale and Obokhare 2009). Studies in mice have observed that constituents of air pollution can cause retarded gonadal cell migration, immature gonads (Tam and Liu 1985), and germ cell differentiation disorders (Yoshida et al. 2002). Two case–control studies of associations between exposure to exhaust fumes and childhood germ cell tumors (all histologic types combined) did not observe associations with exposure to engine exhaust fumes (Chen et al. 2006; Shu et al. 1995); however, the broader age range (< 15 years of age) in these studies suggests they would have included larger numbers of histologic types less frequently observed in young children. In a separate study by our group in which traffic pollution was modeled using land-use regression, we observed positive associations with retinoblastoma and ALL, consistent with the present study, but no associations with germ cell tumors; this study included Los Angeles County residents only, and thus had some overlapping participants with the present study (Ghosh et al. 2013).

PM_2.5_ is an imperfect proxy for traffic-related air pollution. In urban areas, most PM_2.5_ pollution occurs directly or indirectly (via reactions of precursors) from traffic exhaust or other combustion, with much of the remainder from construction dust. In rural areas, dust from agricultural operations and wood smoke make up much larger proportions of PM; and along the California coast, another important contributor to PM is sea salt (Cox et al. 2010). Given that 83% of births in our study occurred in urban areas, gasoline and diesel combustion was the primary contributor to PM_2.5_ exposures for most children.

We observed a negative association between AML and traffic-related pollution. The possibility exists that this result is attributable to chance. Moreover, in California, AML rates are highest across the rural counties of the Sierra Nevada, the Napa Valley, and the North Coast, suggesting that risk factors more common in rural areas may be more relevant for AML etiology (California Cancer Registry 2012). In the present study, AML cases were 20% more likely than controls to live in rural counties. Other studies have similarly reported elevated risks for both adult and childhood AML in rural areas (Carozza et al. 2008; Sinner et al. 2005).

A possible reason for the difference in results between ours and other studies may be that some studies included older children in analyses. A number of childhood cancer studies have posited that risk factors may differ by the age at diagnosis, and have suggested that any effect from pregnancy exposures may be most relevant for younger children (Cnattingius et al. 1995; Heck et al. 2009; Yeazel et al. 1995). When we stratified ALL cases by the age at diagnosis, average CO exposures during pregnancy were more strongly associated with ALL diagnosed during the first year of life than cases diagnosed in older children. Similarly, Savitz and Feingold (1989) reported positive associations between all leukemias and traffic exposure among children < 5 years of age at diagnosis (OR = 5.6; 95% CI: 1.9, 16.7) but no associations among children 5–14 years of age (OR = 0.4; 95% CI: 0.1, 2.8). Vinceti et al. (2012) also reported similar patterns by age.

There are a large number of components of traffic-related air pollution, including thousands of chemicals and particulates, making it difficult to identify the most relevant chemicals for carcinogenesis. Only 50% of benzene emissions in California come from mobile sources, so our CALINE4 model should not be interpreted as a model for benzene exposure (Cox et al. 2010). Because of more stringent requirements, gasoline formulations have changed in the United States in recent years, with consequent decreases in CO emissions observed over the prior two decades. California law has required additional decreases beyond what is allowed in conventional U.S. gasoline, yielding lower emissions of NO_x_ and volatile organic chemicals (U.S. General Accounting Office 2005). Despite this, California is known to have among the highest levels of air pollution in the nation. Of the 25 U.S. metropolitan areas with the highest short-term ambient PM_2.5_ concentrations, 12 are located in California, in part due to the high population density (American Lung Association 2004). In the South Coast Air Basin, where Los Angeles is located, in 2007 PM_2.5_ annually averaged 19.8 μg/m^3^, the maximum 8-hr CO concentration was 5.3 ppm, and the maximum annual average of NO_2_ was 0.031 ppm (Cox et al. 2010). The pollutant values estimated by our CALINE4 model were lower than those typically recorded at monitoring stations in California. The reason for this discrepancy is that CALINE4 models do not consider contributions from minor surface streets, regional background, or other sources such as industrial emissions, minor roads, or traffic emissions from roads > 1,500 m away. Thus, our values will be lower than those typically recorded by air pollution monitors. Because of this, and because modeled CO is a marker of traffic pollution rather than a carcinogen, we calculated ORs based upon the IQR of exposure rather than the absolute exposure level.

Although we recommend the use of complex modeling strategies to estimate exposures whenever possible, simple traffic density might often be the only measure feasible to ascertain exposures in the large populations needed for childhood cancer studies. Traffic density has been computed in various ways across different studies (for example, traffic counts per kilometer of road in an area, sum of the miles of road in an area, traffic counts for major roads only), which may affect the validity of findings and the potential for exposure misclassification.

A limitation of our study was the reliance on the birth certificate for the child’s address and for information on confounding variables. A recent review found that 9–32% of women in the United States and abroad, in studies from the 1980s–2000s, move residence during pregnancy, although most moves are local (median distance, < 10 km) (Bell and Belanger 2012). We also do not know which mothers worked outside the home during pregnancy, which is likely to influence the accuracy of exposure classification. Birth certificates vary in accuracy depending on the information being collected. Validation studies of birth certificates in California and other U.S. states have indicated that factors such as demographic information and parity have a validity of > 90% (Baumeister et al. 2000; Northam and Knapp 2006).

Additionally, there may be neighborhood factors that co-vary with traffic exposure that we could not account for in the present study. In our previous work we did not observe associations between exposure to traffic pollution in Los Angeles and family or neighborhood socioeconomic status (Wilhelm et al. 2009); also associations have not been established between socioeconomic status and most childhood cancer types, with the possible exception of Wilms tumor (Adam et al. 2008; Carozza et al. 2010; de Camargo et al. 2011; Heck et al. 2012; Poole et al. 2006). We have previously estimated correlations between estimated exposures to different types of pollutants in California, and observed that persons exposed to high traffic pollution also tend to be exposed to higher levels of three air toxics: lead, styrene, and perchloroethylene (Heck et al. 2013b). Although none of these three air toxics has been definitively associated with childhood cancers, we cannot exclude that they may contribute to risk.

Strengths of the study include the population-based design and inclusion of rarer childhood cancer types. The population was varied as to its rural/urban area of residence, road density and demographic factors. Our record-linkage approach included all children for whom data were available, limiting selection bias; however, we were limited in being able to estimate only the address at birth.

This study provides new evidence suggesting that exposure to traffic-related pollution in pregnancy and early childhood may increase the risk for ALL, retinoblastoma, and germ cell tumors. Further research using complex air pollution models, examining retinoblastoma and other rarer cancer types, is recommended.

## Correction

The IQR value for PM_2.5_ in Table 2 and the PM_2.5_ values for PNET and medulloblastoma cases in Table 4 were incorrect in the manuscript originally published online. They have been corrected here.
